# Targeting Polo-like kinase 1 in SMARCB1 deleted atypical teratoid rhabdoid tumor

**DOI:** 10.18632/oncotarget.21932

**Published:** 2017-10-19

**Authors:** Irina Alimova, Angela M. Pierce, Peter Harris, Andrew Donson, Diane K. Birks, Eric Prince, Ilango Balakrishnan, Nicholas K. Foreman, Marcel Kool, Lindsey Hoffman, Sujatha Venkataraman, Rajeev Vibhakar

**Affiliations:** ^1^ Department of Pediatrics, University of Colorado Anschutz Medical Campus, Aurora, CO, United States; ^2^ Morgan Adams Foundation Pediatric Brain Tumor Research Program, Children’s Hospital Colorado, Aurora, CO, United States; ^3^ Department of Neurosurgery, University of Colorado Denver, Aurora, CO, United States; ^4^ Division of Pediatric Neurooncology, German Cancer Research Center (DKFZ), Heidelberg, Germany

**Keywords:** Polo-like kinase 1, ATRT, SMARCB1, volasertib

## Abstract

Atypical teratoid rhabdoid tumor (ATRT) is an aggressive and malignant pediatric brain tumor. Polo-like kinase 1 (*PLK1*) is highly expressed in many cancers and essential for mitosis. Overexpression of PLK1 promotes chromosome instability and aneuploidy by overriding the G2-M DNA damage and spindle checkpoints. Recent studies suggest that targeting PLK1 by small molecule inhibitors is a promising approach to tumor therapy. We investigated the effect of PLK1 inhibition in ATRT. Gene expression analysis showed that *PLK1* was overexpressed in ATRT patient samples and tumor cell lines. Genetic inhibition of *PLK1* with shRNA potently suppressed ATRT cell growth *in vitro*. Treatment with the PLK1 inhibitor BI 6727 (Volasertib) significantly decreased cell growth, inhibited clonogenic potential, and induced apoptosis. BI6727 treatment led to G2-M phase arrest, consistent with PLK1’s role as a critical regulator of mitosis. Moreover, inhibition of PLK1 by BI6727 suppressed the tumor-sphere formation of ATRT cells. Treatment also significantly decreased levels of the DNA damage proteins Ku80 and RAD51 and increased γ-H2AX expression, indicating that BI 6727 can induce DNA damage. Importantly, BI6727 significantly enhanced radiation sensitivity of ATRT cells. *In vivo*, BI6727 slowed growth of ATRT tumors and prolonged survival in a xenograft model. PLK1 inhibition is a compelling new therapeutic approach for treating ATRT, and the use of BI6727 should be evaluated in clinical studies.

## INTRODUCTION

ATRT is a highly aggressive brain tumor of childhood with 5-year overall survival of 35% [1, 2]. The salient molecular feature is the loss of the *SMARCB1* gene which results in epigenetic dysregulation of the genome [3, 4]. Recent studies indicate that patients benefit from intensified multimodal therapies but treatment is far from optimal and therapy-related toxicity is a critical problem in this young age group [5]. New therapeutic approaches are critically needed for children with ATRTs.

To address this need we perfomed a combined gene expression analysis along with a RNAi screen to identify potential targets. One key target identified was Polo-like kinase 1 (PLK1). PLK1 is a serine/threonine kinase that plays important roles in aspects of mitosis, including centrosome maturation, mitotic entry, chromosome segregation, and cytokinesis [6, 7]. PLK1 promotes mitotic entry by phosphorylating cyclin B1 and CDK1, and it initiates mitotic exit by activating the anaphase promoting complex [6]. PLK1 is essential for DNA damage response, cell cycle division, and spindle formation [8]. Overexpression of PLK1 leads to chromosome instability and aneuploidy by overriding the G2-M DNA damage and spindle checkpoints [6]. Deactivation of PLK1 with shRNA or small molecule inhibitors decreases cell proliferation *in vitro* and *in vivo* [6, 9]. Because PLK1 has been preclinically validated as a cancer therapeutic target, small-molecule inhibitors of PLK1 have become attractive candidates for treating cancers such as rhabdomyosarcoma and neuroblastoma [10-12]. We and others have demonstrated that PLK1 is a potential therapeutic target for brain tumors such as glioblastoma, medulloblastoma, and diffuse intrinsic pontine glioma (DIPG) [13-16].

In this study, we investigated whether PLK1 is a therapeutic target for ATRT. We used a highly selective and potent PLK1 inhibitor, volasertib (BI 6727), which acts as an ATP-competitive kinase and has broad antitumor activity in preclinical and clinical studies [17-22]. We investigated the effects of BI 6727 on ATRT alone or in combination with radiotherapy.

## RESULTS

### *PLK1* is overexpressed in ATRT patient samples and ATRT cell lines

**Figure 1:**
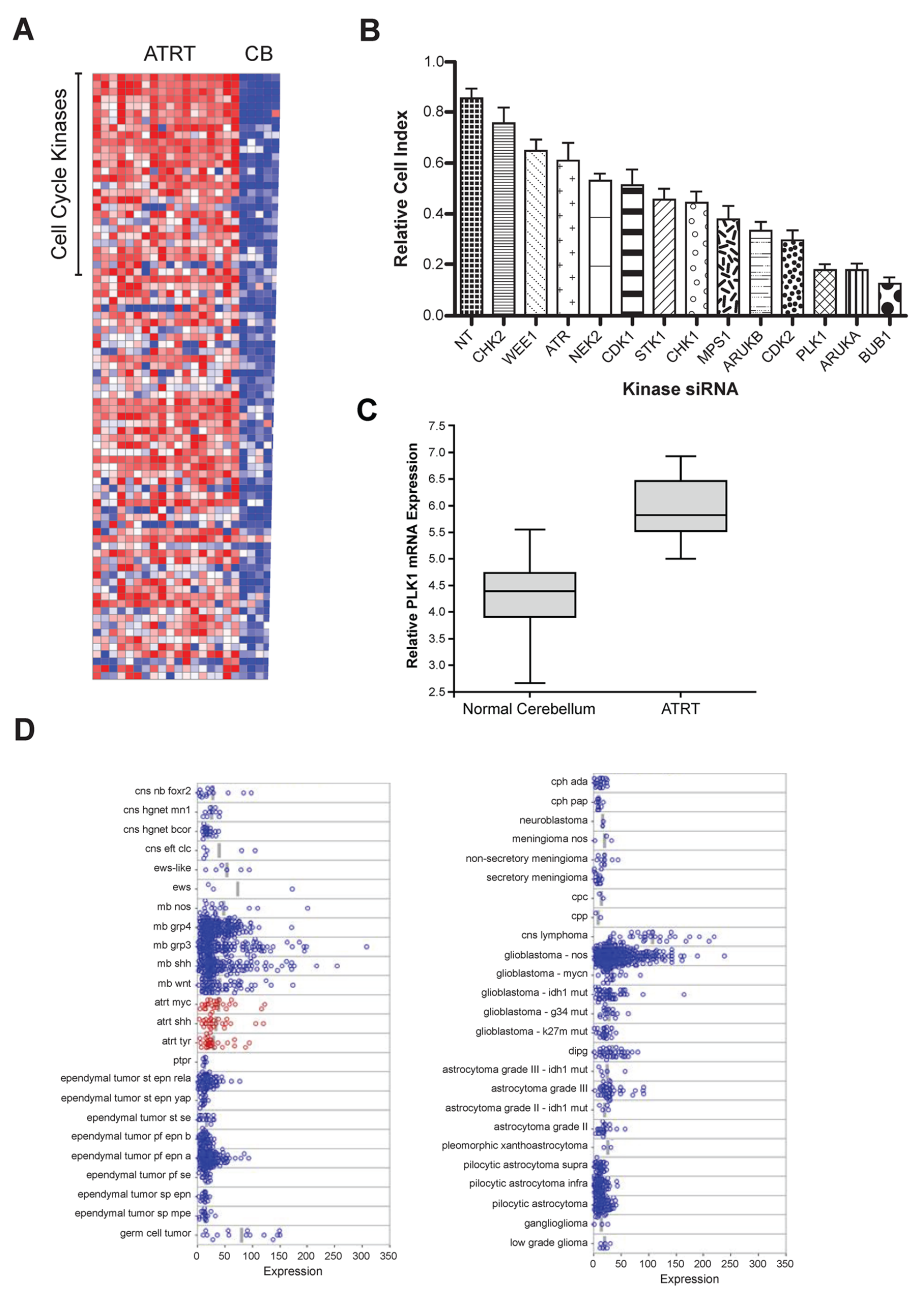
Microarray gene expression analysis and a genome-wide RNAi screens for kinases regulating ATRT cell proliferation: PLK1 identified as a potential target **(A)** Expression of kinases in a group of ATRT patient samples compared to that in normal cerebellum [24] using a heat map, where red indicates increased expression and blue indicates repressed expression. In ATRT samples, G2-M phase cell cycle regulated kinases are highly expressed and can be a potential therapeutic targets in ATRT. **(B)** By genome-wide RNAi screening in ATRT cell lines, top 10% of the kinases that are crucial for ATRT cell proliferation were identified. To further validate, shRNAs were used to knock down each of those top genes in ATRT cell lines and performed cell proliferation assay using XCELLigence system. Consistent with the primary screen hits, knockdown of PLK1, AURKA and BUB1 decreased the ATRT cell proliferation significantly. **(C)** In a cohort of ATRT patient samples, PLK1 mRNA expression was significantly elevated when compared to normal cerebellum. **(D)** Microarray data analysis of PLK1 expression in multiple pediatric brain tumors in a larger cohort of patient samples. PLK1 is overexpressed in all three sub-groups of ATRT tumors.

Kinases involved in cell cycle division and mitotic progression are very promising targets for cancer therapy [6, 9, 23-25]. To identify kinases that might be therapeutic targets for ATRT, we measured the mRNA expression level of all kinases in brain samples from ATRT patients (n=18) and healthy controls (n=5) (Figure 1A). Many cell cycle kinases were significantly overexpressed in ATRT patient samples, suggesting that they may play a role in ATRT tumorigenesis. Most of the affected kinases are active at the G2-M checkpoint. To determine which kinases were most important for ATRT growth, we performed a small siRNA screen (Figure 1B) to knock down the expression of the top G2-M phase genes. *PLK1* was one of the most potent targets (Figure 1B). Importantly Aurora Kinase A was a significant hit, consistent with our prior data demonstrating the role of Aurora Kinase A in ATRT [26]. Based on this result and the overexpression of *PLK1* in childhood cancers [14, 16], PLK1 was selected for further evaluation. Importantly Morozov et al previously showed that PLK1 mRNA was down regulated by Interferon treatment of SMARCB1 deficient cells [27].

Expression of PLK1 mRNA was significantly higher than normal cerebellum in a small cohort of patient samples from the Children’s hospital of Colorado (Figure 1C). Given that the cell of origin for ATRT is not known we used normal cerebellum as a comparator. These data were further confirmed in a large database of tumor samples (Figure 1D) [28]. Interestingly as previously described many brain tumors exhibit high PLK1 mRNA expression including Glioblastoma and Medulloblastoma [13, 14]. Next, PLK1 protein levels in ATRT cells were analyzed in two well-characterized ATRT cell lines (BT12 and BT16) and one primary short-term culture from a patient with ATRT (MAF-A737). PLK1 protein was higher in all cell lines than in normal cerebellar tissue (Supplementary Figure 1A). In addition, quantitative reverse-transcriptase PCR confirmed PLK1 overexpression in ATRT cell lines (Supplementary Figure 1B). None of the three cell lines expressed the SMARCB1/INI1 protein (Supplementary Figure 1C).

### Genetic inhibition of PLK1 decreases clonogenic potential of ATRT cells

**Figure 2:**
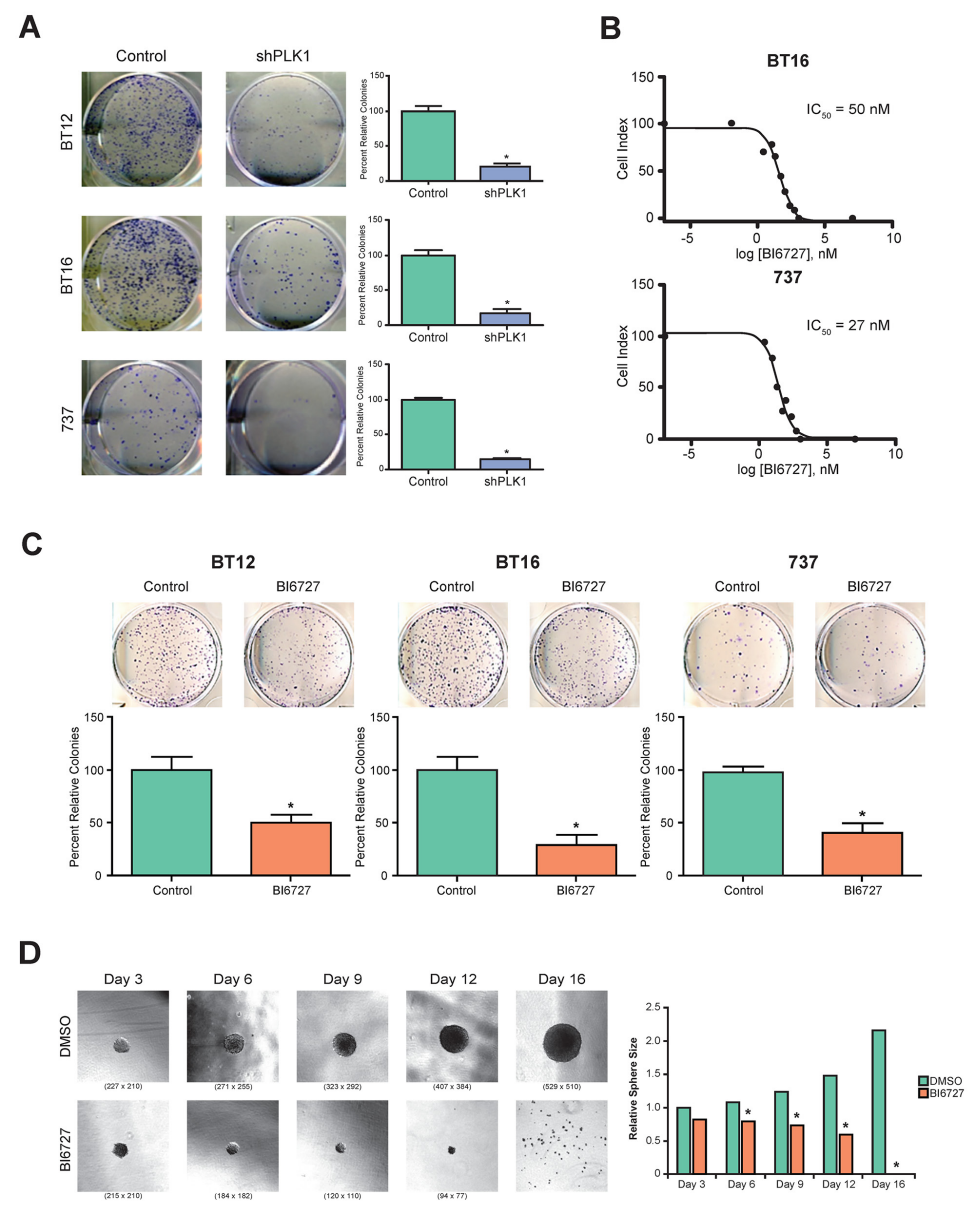
Genetic and pharmacological inhibition of PLK1 in ATRT cell lines: tumor cell growth and tumor-sphere formation was inhibited **(A)** Three ATRT cell lines, BT12, BT16 and MAF-A737, were used to study the effect of PLK1 inhibition using shRNA. Knockdown of PLK1 significantly decreased the tumor cell colony forming efficiency in all three ATRT cell lines (p< 0.05 shPLK1 vs. control (shNULL)). **(B)** BI6727 (Volasertib), a specific inhibitor of PLK1 was used to inhibit PLK1 expression in ATRT cell lines. Various concentrations of BI6727 were used to identify IC_50_ of the drug in all three ATRT cell lines and cell proliferation was measured by XCELLigence system. The representative IC_50_ graph for BT16 and 737 cell lines were shown. **(C)** The three ATRT cell lines, BT12, BT16 and MAF-A737, treated with the IC30 concentration of BI6727, 30 nM, and 14.5 nM respectively, showed marked decrease in cell colony forming ability. Representative images and the quantification of colonies formed were shown. ^*^p < 0.05 control (DMSO) vs. BI6727 treatment, ±SEM. **(D)** BI6727 suppresses the tumor sphere formation of ATRT cell line, MAF-A737. The average diameter of the neurospheres decreased with IC_50_ of BI6727 treatment after 3, 6, 9,12 and 16 days of culture. Bright field images of neurospheres after indicated days of culture in the neurosphere media with bFGF and EGF are shown. Quantitation of average neurosphere diameter was shown in bar graph (^*^p <0.05 BI6727 vs Control, DMSO).

In a previous study, we demonstrated that reducing levels of PLK1 with RNAi significantly decreased the ability of medulloblastoma cells to form colonies [14]. To determine whether PLK1 is important for ATRT tumorigenesis, BT12, BT16, and MAF-A737 cell lines were transfected with vector control shNull or shPLK1 and seeded for colony formation assay. The colonies were counted and plotted. PLK1 reduction by RNAi significantly decreased colony formation for all cell lines (Figure 2A; *P* < 0.05).

### Pharmacologic inhibition of PLK1 suppresses ATRT cell growth and abrogates clonogenicity of tumor cells

Recently, BI 6727 was found to inhibit PLK1 activity selectively in many cancers [16, 18, 21, 29]. We evaluated whether BI 6727 would alter ATRT cell growth. BT16 and MAF-A737 cells were treated with varying concentrations of BI 6727, and cell growth was evaluated by an xCelligence assay. BI 6727 decreased ATRT cell growth with an IC_50_ of 50 nM and an IC_50_ of 27 nM for BT16 and MAF-A737, respectively (Figure 2B; Supplementary Figure 2). Moreover, BI 6727 abrogated clonogenicity of ATRT cells (Figure 2C). The sensitivity of ATRT cells to PLK1 inhibition by BI 6727 is similar to sensitivity of medulloblastoma cells and DIPG cells as previously described by us [14].

### BI 6727 treatment reduces the self-renewal of ATRT cells

We previously showed that PLK1 plays a key role in regulating stem-like characteristics of medulloblastoma cells [14]. To determine whether PLK1 is similarly necessary for ATRT-initiating cells, we examined the effects of BI 6727 on the ability of ATRT cells to form tumor spheres. MAF-A737 cells were cultured in stem cell media (neurobasal with epidermal growth factor and leukemia inhibitory factor). After small sphere formation was established, treatment with BI 6727 was initiated, and spheres allowed to form for an additional 16 days. By day 6, sphere diameter in the BI 6727-treated group was significantly decreased (Figure 2D; *P* < 0.05). By day 16, all spheres in the treated group were depleted.

### Inhibition of PLK1 with BI 6727 decreases S phase length and induces G2/M phase arrest in ATRT Cells

**Figure 3:**
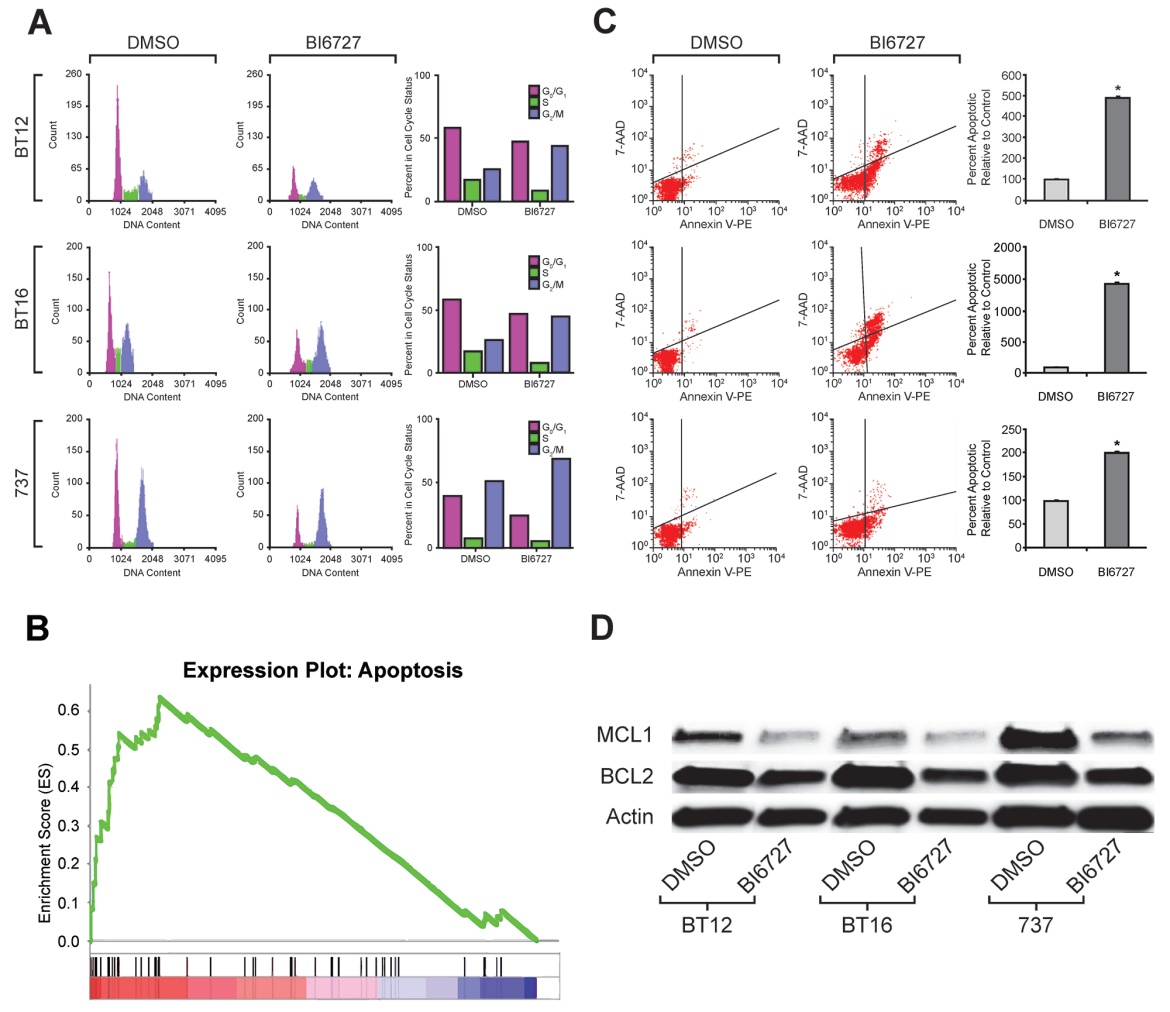
Effect of PLK1 inhibition on cell cycle and cell apoptosis: induced G2/M arrest and increased cell death **(A)** FACS analysis shows increased G2/M arrest that was seen consistently in all three ATRT cell lines treated with their IC_50_ concentrations of BI6727 for 72 hours and analyzed with flow cytometer. There was a concomitant decrease in S phase length as shown. The experiment was done in triplicate and representative flow plots and quantitation were shown. **(B)** GSEA analysis from RNA-seq data showing increased apoptotic gene set signatures in ATRT cell lines treated with IC_50_ of BI6727 compared to that of control, DMSO treatment. Normalized Enrichment Scores (NES) = 1.97 (p<0.001). **(C)** The three ATRT cell lines were treated with their respective IC_50_ of BI6727 for 72 h and apoptosis was measured using Annexin V FITC reagent. Bar chart showing increased proportion of apoptotic cells after BI6727 treatment. The representative results of three independent experiments are shown. *p <0.05 BI6727 vs. DMSO. **(D)** Western blot analysis showing the down-regulation of two major proteins that rescue tumor cells from apoptotic cell death, MCL1 and BCL2, when the three ATRT cell lines shown above were treated with their respective IC_50_ of BI6727.

To evaluate the impact of PLK1 inhibition on ATRT cells further, flow cytometric cell cycle analysis was performed. Cells were treated with 50 nM (BT16 and BT12) or 27 nM (MAF-A737) BI 6727 for 72 hours. Cell cycle analysis demonstrated a reduction in the S phase fraction in response to BI 6727 treatment in all tested cells. After 72 hours of treatment, BI 6727 decreased DNA synthesis (S phase fraction) by 4–9% and increased the percentage of cells in G2/M phase by 20% in all cell lines tested (*P* < 0.05; Figure 3A, Supplementary Figure 3A-C).

### BI 6727 treatment of ATRT cells alters apoptotic gene expression and induces apoptosis

To investigate the transcriptional effect of BI 6727 treatment, we performed gene set enrichment analysis (GSEA) on gene expression data from MAF-A737 cells treated with BI 6727 compared to matched primary tumor gene expression microarray data. Genes involved in apoptotic pathways were significantly increased in MAF-A737 cells after BI 6727 treatment (NES=1.97, *P* < 0.001, Figure 3B). In addition to cell cycle alterations, BI 6727 treatment increased apoptosis after either 24 hours (BT16 cells) or 48 hours (MAF-A737 cells). Apoptosis was significantly increased in all tested ATRT cells after 72 hours of treatment (*P* < 0.05; Figure 3C, Supplementary Figure 4). Because the BCL -2 protein family controls the commitment of cells to apoptosis [30], the effect of BI 6727 treatment on BCL-2 family members was evaluated. ATRT cells were treated with BI 6727 for 72 hours, and treatment decreased the pro-survival family proteins MCL1 and BCL-2 (Figure 3D, Supplementary Figure 5).

### BI 6727 treatment of ATRT cell lines causes DNA damage and strongly decreases DNA repair gene expression

**Figure 4:**
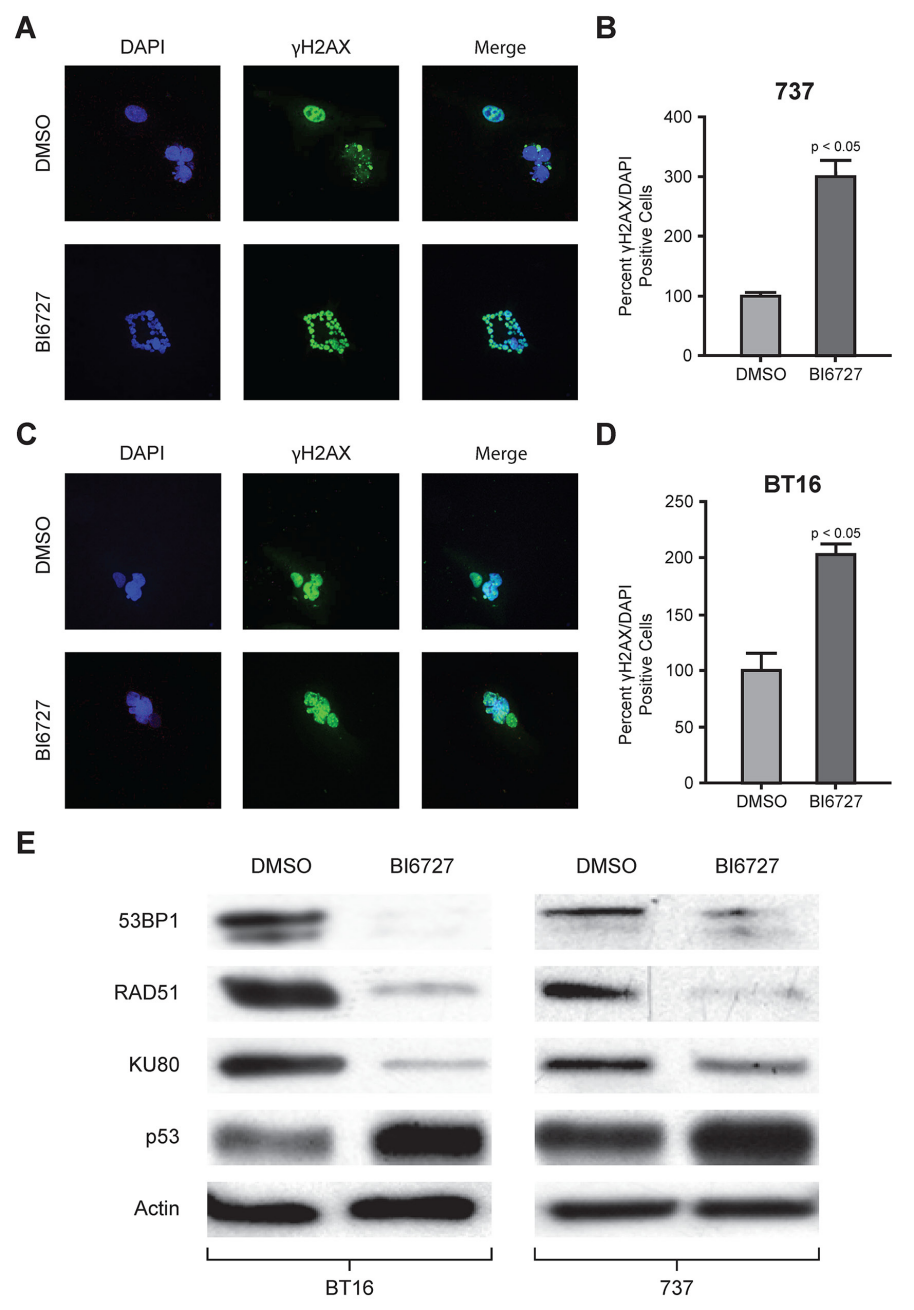
Immunofluorescence analysis of γH2AX and western blot analysis of DNA repair protein with BI6727 treatment: reduction in DNA repair proteins with increased DNA damage and increased tumor suppressor protein levels ATRT cell lines, MAF-A737 and BT16, both were treated with their respective IC_50_ of BI6727 or DMSO for 72 h. Cells were fixed and stained for γH2AX (green) and the nuclei stained with DAPI (blue). Representative immunofluorescent images were shown; MAF-A737 **(A)** and BT16 **(C)**. Quantification of γH2AX foci at 72 h after BI6727 treatment normalized to DAPI was shown for each of the two cell lines. Percent± SEM is shown **(B** and **D)**. **(E)** Lysates from ATRT cell lines treated with either DMSO or with IC_50_ of BI6727 were analyzed by western blotting for DNA damage repair proteins, 53BP1, RAD51 and KU80 along with tumor suppressor protein p53.

To evaluate the DNA damage response due to PLK1 inhibition, ATRT cells were treated with BI 6727 and co-stained with γ-H2AX and DAPI (Figure 4A and 4C).γ-H2AX is a surrogate marker for DNA damage that is recruited to sites of damaged DNA and then removed once DNA is repaired. Immunofluorescence was quantified using Image J (https://imagej.nih.gov/ij). Quantification of the percent of γ-H2AX positive cells was normalized to DAPI. After 72 hours, treatment with BI 6727 induced DNA damage in both MAF-A737 and BT16 cells (Figure 4B and 4D). The increase in γ-H2AX foci was statistically significant (*P* < 0.05). To investigate induction of DNA damage further, we analyzed the expression of proteins associated with DNA damage repair and apoptosis. BT16 and MAF-A737 cells were treated with BI 6727 for 72 hours, and expression of DNA damage response proteins and apoptotic proteins was evaluated by western blot. PLK1 inhibition strongly decreased DNA repair genes, such as *53BP1*, *Ku80*, and *RAD51*, and induced expression of p53, further confirming the induction of DNA damage in response to PLK1 inhibition (Figure 4E).

### BI 6727 pretreatment enhances radiation sensitivity of ATRT cells

**Figure 5:**
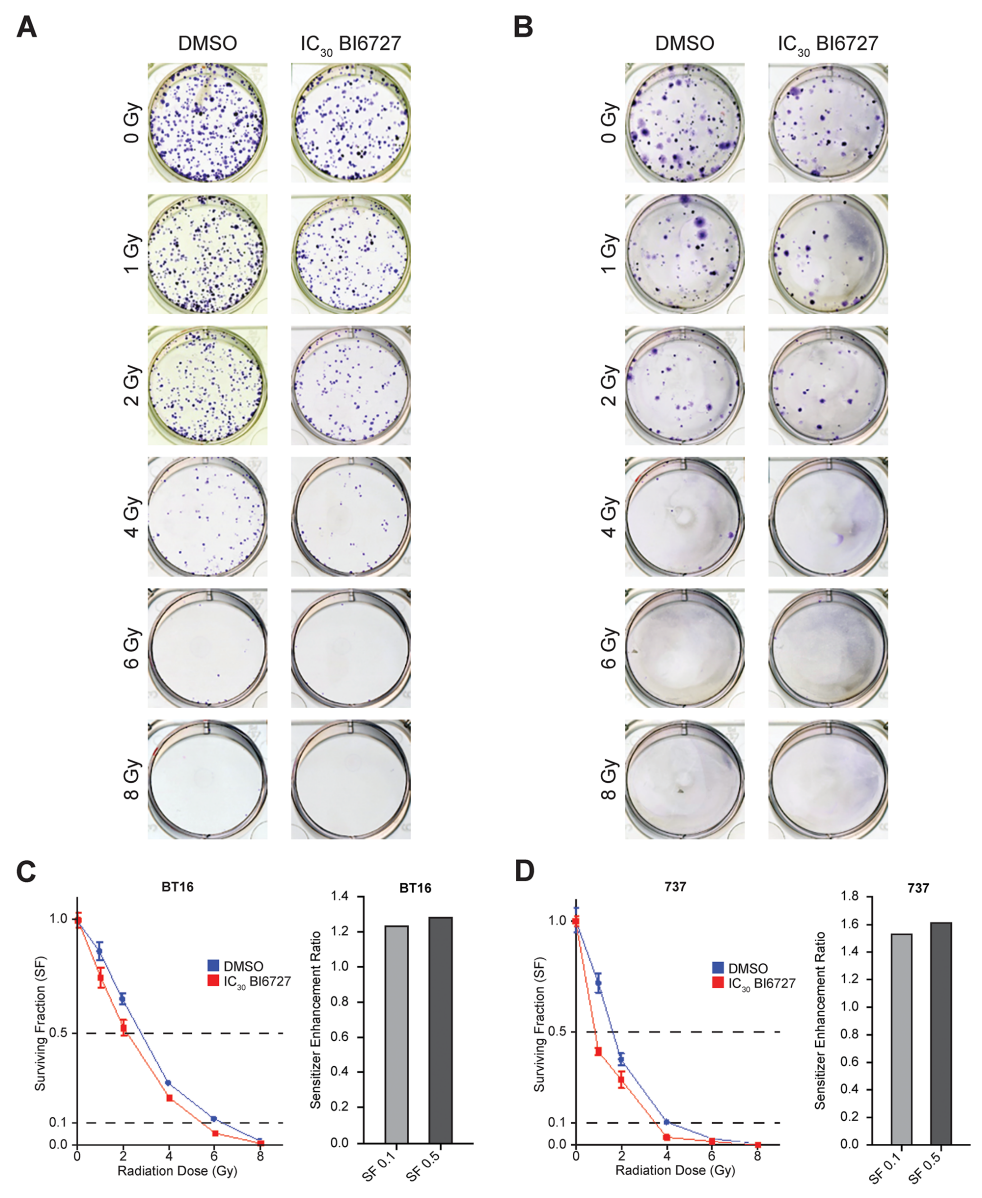
Clonogenic survival results for ATRT cell lines treated with radiation ± BI6727: increased radiosensitization of ATRT cells with BI6727 treatment **(A&B)** Representative well-images of colonies formed when BT16 (A) and MAF-A737 (B) cell lines were pre-treated with IC_50_ of BI6727 and exposed to different radiation doses. **(C&D)** Numbers of colonies with more than 40 cells were counted and cell surviving fraction was plotted. BT16 (C) and MAF-A737 (D): DMSO control (blue line) and BI6727 treated (red line). Bar graphs on the side show the sensitivity enhancement ratio (SER) for each cell lines calculated at the 10% (SF 0.1) and 50% (SF 0.5) survival.

To investigate whether PLK1 inhibition enhances radiation sensitivity of ATRT cells, we treated BT16 and MAF-A737 ATRT cells with BI 6727 for 24 hours, irradiated them with different doses of radiation, and conducted a colony formation assay (Figure 5A and 5B). We calculated the surviving fraction (SF) of cells treated with DMSO or BI 6727 across a range of radiation doses. Pretreatment with BI 6727 decreased the SF in response to radiation for both BT16 and MAF-A737 cells (Figure 5C and 5D). At 2 Gy irradiation and higher, the surviving fraction of BI 6727 pretreated cells was significantly lower than that of untreated cells. Sensitized enhancement ratios (SER) were calculated from surviving fractions of BI 6727-treated and DMSO treated cells. We calculated SER at 10% and 50% survival. Any SER value greater than one indicates enhancement of radiation. The SERs were 1.24 at 10% survival fraction and 1.28 at 50% survival fraction for BT16 cells and 1.54 and 1.62 for MAF-A737 cells, demonstrating a strong potentiation of the effects of radiation after PLK1 inhibition (Figure 5C and 5D). Thus, BI 6727 pretreatment sensitized human ATRT cells to ionizing radiation in both BT16 and MAF-A737 cells. These data establish the concept that PLK1 inhibition is a potent radio-sensitization strategy in ATRT.

### BI 6727 inhibits tumor growth *in vivo*

**Figure 6:**
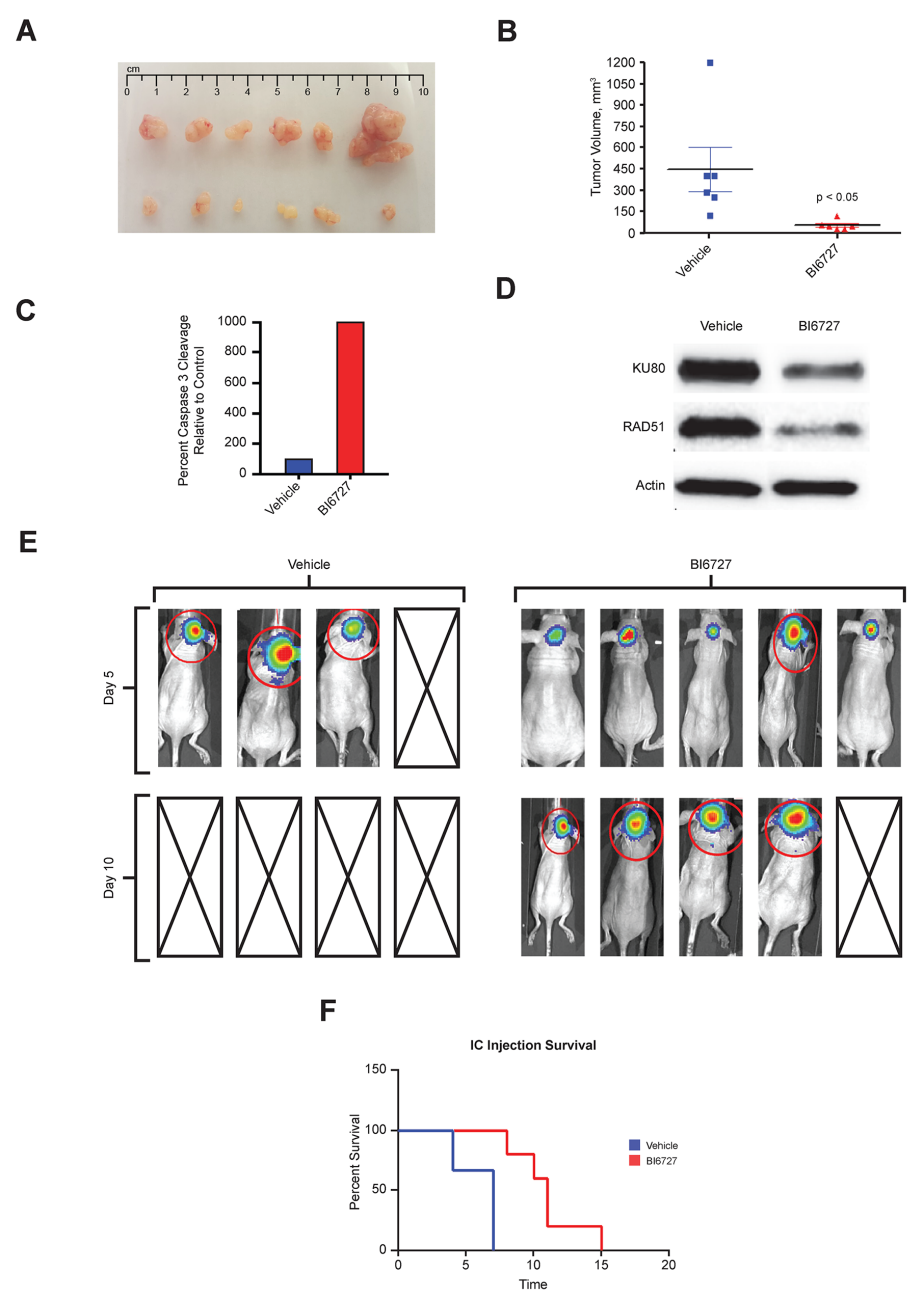
*In Vivo* efficacy of BI6727 in pediatric ATRT mouse xenografts: decreased tumor burden and increased survival **(A)** Tumors dissected from subcutaneously injected luciferase-expressing BT16 cells: Vehicle treatment (top row), BI6727 treatment with 25mg/kg, 2 days per week for 3 weeks by oral gavage (bottom row). **(B)** Quantification of tumor size in BI6727 treated animals compared to vehicle (p<0.05). **(C)** Quantification of cleaved caspase 3 staining by IHC in BT16 mouse xenografts after drug treatment. **(D)** Protein lysates from the BT16 xenograft tumor tissue by western blotting showing RAD51 and Ku80 expression. **(E)** Heat map image representations of bioluminescence intensity after intracranial injection of BT16 cells for mice on day 5 and on day 10 after treatment: control (left), BI6727 (right) and boxes with diagonal lines denoting mouse sacrificed during experiments as they reached the end point. **(F)** Kaplan-Meier survival curve of BI6727-treated and Vehicle-DMSO treated tumor bearing mice (n= 8 to 10 mice/group). BI6727 increased survival of BI6727 treated mice (p<0.05).

To examine the effect of BI 6727 *in vivo*, luciferase-expressing BT16 ATRT cells were injected in the flank of nude mice. After tumor growth was detected, mice were given vehicle or 25 mg/kg of BI 6727 by oral gavage on two consecutive days per week for 3 weeks. The mice were sacrificed, and the tumors were collected and measured. Figure 6A shows representative images of tumors in treated and untreated animals. BI 6727 significantly decreased tumor size in treated animals (*P* < 0.05; Figure 6B). Cleaved Caspase 3, a marker of apoptosis, was increased in BI 6727 treated xenografts (Figure 6C). Moreover, BI 6727 strongly decreased expression of the DNA repair genes Ku80 and *RAD51* (Figure 6D, Supplementary Figure 6).

To evaluate a biologically relevant model we generated an orthotopic ATRT xenograft in murine cerebella. In this intracranial mouse model, BI 6727 treatment nearly doubled survival compared to untreated controls. Median survival of the control group was 6 days, compared to 11 days for BI 6727-treated mice (*P* < 0.05; Figure 6F). Five days after beginning therapy BI6727 treated animals showed smaller tumors compared to vehicle controls (Figure 6E). By 10 days of treatment all the vehicle- treated animals were deceased while the BI6727-treated animals were alive (Figure 6E and 6F). In addition, the proliferation marker Ki-67 was lower in treated tumors than in controls (Supplementary Figure 7B). Tumors had a *SMARCB1/INI1* deletion and were negative for INI1 (Supplementary Figure 7A).

## DISCUSSION

ATRT is an aggressive, rare, malignant brain tumor that occurs mainly in early childhood [31]. Treatment relies on highly toxic chemotherapy and radiotherapy [2, 32]. To improve outcomes and decrease morbidity, more targeted therapy is required. Here we describe a combined approach using genomic data with functional RNAi screening to identify PLK1 as a novel therapeutic target in ATRT.

*PLK1* has previously been implicated in several adult and pediatric cancers including CNS tumors [11, 12, 14, 16, 25]. Here we show that PLK1 expression is increased in ATRT tissues and is a potential target for treatment. Blocking the expression of *PLK1* by shRNA suppressed the growth of ATRT cells and reduced clonogenicity. Previous studies show that Interferon can regulate PLK1 expression in SMARCB1 deleted cells and drive senescence further emphasizing the role of PLK1 in ATRT [27].

Recently we and others have demonstrated that targeting PLK1 with the small molecule inhibitors BI 2536 [14] and BI 6727 [16, 17, 22, 33, 34] reduced cancer cell proliferation. BI 6727, developed as a second-in-class dihydropteridinone derivative, and has a better pharmacological profile than its predecessor BI 2536 [17, 34]. BI 6727 has demonstrated clinical efficacy in multiple tumor types [16, 17, 18, 19, 25, 30, 33]. We show that treating cells with clinically relevant doses of BI 6727 resulted in a marked reduction of cell proliferation and tumor cell clonogenicity. Our data show that BI6727 increased S-phase length and G2-M phase arrest correlate with our previous findings, confirming that PLK1 plays very important role in cell cycle regulation [14, 16]. Importantly we determined that BI 6727 decreases pro-apoptotic signaling including suppression of BCL-2 family members such as MCL1 and found that MCL1 levels were also reduced in treated mouse tumors, suggesting that MCL1 protein levels might be an interesting biomarker of BI 6727 response. Further investigations are needed to evaluate this possibility.

In our ATRT xenograft mouse model, analysis of cleaved caspase 3 demonstrated that BI 6727 treatment induced apoptosis

Current ATRT therapy consists of intensive chemotherapy in combination with radiation [32, 35], but new therapies would ideally improve outcomes while decreasing toxicity. Thus, agents that radiosensitize ATRT cells would be valuable. We found that low nanomolar concentrations of BI 6727 can effectively enhance ATRT cell radiosensitivity *in vitro*. Our findings correlate with previous studies showing increased radiosensitivity in malignant cells depleted of PLK1 by RNAi or by small molecule inhibitors [14, 16, 35-40].

Our data, together with previous studies, demonstrate that PLK1 is a compelling potential target in many cancers, including ATRT. BI 6727 is a highly effective PLK1 inhibitor, causing tumor regression in our ATRT model.

In summary, we have demonstrated that a mouse model of ATRT can be successfully treated with chemical or genetic PLK1 inhibition. Moreover, in combination with radiation therapy, PLK1 inhibition is a promising strategy for treatment of ATRT in humans. While a recent study of BI6727 (Volesertib) demonstrated disappointing results in a phase three trial of adult AML patients we suggest that BI 6727 and other PLK1 inhibitors are excellent candidates for treatment of ATRT and should undergo further clinical investigation in children. Thus we would suggest that PLK1 is an attractive target for ATRT therapy and that BI6727 is an excellent agent to pursue such strategies.

## MATERIALS AND METHODS

### Cell lines and reagents

The small molecule PLK1 inhibitor BI6727 was purchased from Chemitek and from Selleckchem. The drugs were reconstituted in dimethyl sulfoxide (DMSO). An equivalent amount of DMSO for the highest concentration of drug was used for each experiment as a vehicle control. Antibodies used for western blot analysis were from the following sources: actin #8H10D10, p21#2947, Rad 51#8875, 53BP1#4937, Plk1#5472, ki67# 9449, KU80#2753, Phospho-H2AX#2577 were purchased from Cell Signaling, USA; p53 ab179477 from abcam, INI-1(Anti-BAF47) from BD Biosciences, USA. BT12, BT16 atypical teratoid/rhabdoid tumor (ATRT) cell lines were kindly provided by Dr. Peter Houghton’s laboratory (St. Jude Children’s Research Hospital). The short-term ATRT cell culture (MAF-A737) was established from a surgical sample obtained from Children’s Hospital Colorado under an approved Institutional Review Board [41] protocol. Cells were cultured in RPMI medium (Gibco, Grand Island, NY) supplemented with 10% fetal bovine serum (FBS, Atlanta Biologicals, Lawrenceville, GA) according to the supplier’s recommendations.

Normal brain tissue was collected from autopsy or purchased from Ambion (Austin, TX), Stratagene (Santa Clara, CA) and Clontech Laboratories, Inc. (Mountain View, CA). Primary patient samples were obtained from Children’s Hospital Colorado and were conducted in accordance with local and federal human research protection guidelines and Institutional Review Board regulations [41]. Informed consent was obtained for all specimens collected.

### Quantitative real-time polymerase chain reaction

Ribonucleic acid was isolated 48 hours after transfection using a Qiagen RNeasy kit (Valencia, CA). TaqMan gene expression primers and probes for *PLK1* (Hs00153444_m1), and *GAPDH* (Hs99999905_m1) were purchased from Applied Biosystems (Carlsbad, CA). Assays were performed in triplicate according to the manufacturer’s recommendations. *GAPDH* was used as an endogenous control and the gene expression relative quantity was calculated using the ΔΔC_t_ method. Gene expression assays were performed on an ABI StepOnePlus Real-Time PCR system.

### Transfection of ATRT cells with shPLK1

shRNA vector targeting *PLK1* mRNA (1325: CCGGCCCGAGGTGCTGAGCAAGAAACTCGAGTTTCTTGCTCAGCACCTCGGGTTTTTG) and a non-targeting shRNA (Control) were purchased from the Functional Genomics Facility at the University of Colorado, Boulder, and transfected into ATRT cell lines using the Lipofectamine 2000 Transfection Reagent (Invitrogen, Carlsbad, CA). One microgram of shRNA for a 6-well plate was transfected into ATRT cell lines. The ratio used for the forward transfections was 1 microgram of shRNA DNA: 2 μl of Lipofectamine 2000 Transfection Reagent.

### Colony formation assay

Control or transfected cells were seeded into 6-well plates in triplicate at density of 500 cells/well in 3 ml medium containing 10% FBS. Cells were grown for 10 days. The cell clones were visualized by staining with crystal violet. The colony numbers (>50 cells per colony) were counted using a electronic counter (Heathrow Scientific, Vernon Hills, IL) and inverted microscope.

### Cell proliferation

Cell proliferation (cell index) was measured on the XCELLigence Real-Time Cell Analyzer instrument (Roche). Cells were seeded in triplicate at 1000 cells/well in the E-Plate 96, followed by drug treament 24 hour later. Cell growth was monitored for 4 days.

### Tumor spheres assay

MAF-A737 neurospheres were allowed to form in a 24 well ultra-low cluster plate (500 cells per well in triplicate) in serum free media (neurobasal with epidermal growth factor and leukemia inhibitory factor). After cells formed spheres (72hrs) they were treated with BI6727 (IC50=24 nm for MAF-A737). Sphere size was measured on day 3, 6, 9, 12 and 16 after BI6727 treatment.

### Cell cycle analysis

Flow cytometric analysis was performed on the Guava EasyCyte Plus flow cytometer (Millipore). Cells were seeded in 6-well plates (10^5^ cells/well) and trated 24 hrs later with an IC 50 of BI6727. Cells were harvested 48 and 72 hours later by trypsinization and fixed with chilled 70% ethanol for 24 hrs. Fixed cells were then washed and stained with propodium iodide-containing cell cycle reagent (Millipore).

### Gene expression microarray analysis

Patient tumor samples were evaluated for gene expression using Affymetrix U133 Plus 2.0 GeneChip microarrays. Briefly, samples were snap-frozen in liquid nitrogen at the time of surgery. An RNeasy kit (Qiagen, Valencia, CA) was used to extract ribonucleic acid from each sample using. Samples were hybridized to HGU133 Plus 2.0 GeneChips (Affymetrix, Santa Clara, CA) according to the manufacturer’s instructions. All microarray data from the samples was background-corrected and normalized using the gcRMA algorithm. One probe set per gene was selected for use in subsequent analyses based on highest overall expression level across samples. Normalized Enrichment Score (NES) was used to identify genes in response to BI 6727 treatment. Differential expression of genes was determined using a Student’s t-test.

### Cell apoptosis assay

Cells were treated with BI 6727 (BT16 IC50=50nm; BT12 and MAF-A737 IC50= 24 nm), and then allowed to grow in normal culture medium for 24, 48 and 72 hours. The cell concentration was determined following staining with Guava ViaCount reagent (Millipore, Billerica, MA). Equal numbers of cells were then stained using Guava Nexin reagent (Millipore) to detect apoptotic cells. Samples were run on a Guava EasyCyte Plus flow cytometer (Millipore).

### Western blotting

Protein expression levels were determined by western blotting as previously described by us. Equal amounts of cell lysates were resolved by sodium dodecyl sulfate polyacrylamide gel electrophoresis (SDS-PAGE), and western blot analysis was performed with specific antibodies as described above.

### Immunofluorescence

Three thousand MAF-A737 cells grown in poly-D-lysine coated chamber slides were treated with an IC50 of BI6727 or DMSO for 72 hours. After treatment cells were washed and fixed with 4% paraformaldehyde for 15 minutes at room temperature. Cells were then permeabilized with 0.2% Triton X-100 in PBS for 15 minutes followed by incubation in 5% milk diluted in 0.05% Triton X-100 for 30 minutes at room temperature on a shaker. After blocking, cells were incubated with the primary antibodies. The following antibodies were used at a dilution of 1:200, γH2AX for 1 hour at room temperature. After washing with 0.05% Triton X-100 (3 times for 5 minutes each) cells were incubated with Alexa Fluor 488 conjugated secondary antibody (1:500) for 1 hour at room temperature in the dark, washed with PBS (3 times for 5 minutes each) and mounted using ProLong Gold antifade reagent containing DAPI (Sigma). Images were acquired using an inverted epifluorescence microscope at a magnification of 20x. At least 10 random fields were chosen to count cells containing greater than 10 foci.

### Combination of BI 6727 and ionizing radiation

Five hundred cells per well of a 6-well plate were plated in triplicate for 24 hours before addition of BI 6727. Cells were exposed to drug for 24 hours, and then drug-containing medium was aspirated and normal culture medium was added. Cells were then immediately irradiated. After eight days of additional growth, wells were stained with crystal violet solution and colonies were counted as described above. Survival curves were generated after normalizing for BI 6727-induced death. Non-linear regressions were calculated for each line. The radiation dose intersecting the non-linear regression for a 10% (SF0.1) and 50% (SF0.5) surviving fraction was calculated for each drug dose. The sensitizer enhancement ratio (SER) was then calculated as follows:

SER= SFx for IR + DMSO/ SFx for IR + BI6727

### *In vivo* xenograft experiments

For orthotopic (intracranial) xenograft assays, female athymic nude (*Foxn1*^*nu*^) mice (Envigo, Indianapolis, IN) age 4-12 weeks were anesthetized with isoflurane (VetOne, MWI Boise, ID) and immobilized in a stereotaxic apparatus (David Kopf Instruments, Tujunga, CA). An incision was made in the skin of the skull to expose lambda and a small burr hole was drilled into the skull at 1.5 mm lateral and 2.0 mm posterior to lambda using a Dremel drill (Racine, WI) and a 1.00 mm dental drill bit (Stoelting, Wood Dale, IL). Luciferase-expressing BT16 ATRT cells (5 × 10^5^ in 3 μl serum-free medium) were injected 3.0 mm below the surface of the skull into the cerebellum at a rate of 600 nl/min using a MicroUltra Pump (World Precision Instruments, Inc., Sarasota, FL) fitted with a 10 ul Hamilton syringe and 26 G needle (Hamilton Company, Reno, NV). The burr hole was sealed with bone wax (World Precision Instruments, Inc., Sarasota, FL) and the incision closed with sterile absorbable suture (Ethicon, Somerville, NJ). Mice were treated with either vehicle (0.5% hydroxyethyl cellulose; Amresco, Solon, OH) or 25 mg/kg BI6727) in vehicle two consecutive days per week by oral gavage. For flank xenograft studies, luciferase-expressing BT16 ATRT cells (3 × 10^6^ in 150 μl serum-free medium) were injected subcutaneously into the flank of athymic mice. Once tumors were palpable and detected on IVIS, mice were treated with BI6727 or vehicle as described above. To monitor tumor growth, mice were injected intraperitoneally with 10 ul/g of 15 mg/mL D-luciferin potassium salt solution (Gold Biotechnology, St. Louis, MO) and imaged using the Xenogen IVIS 200 *In Vivo* Imaging System (PerkinElmer, Waltham, MA). Tumor bioluminescence was analyzed using Living Image 2.60.1 software (Caliper Life Sciences, PerkinElmer, Waltham, MA). Animal care and experimental procedures were conducted in accordance with the guidelines of the University of Colorado Center for Comparative Medicine and the University of Colorado Institutional Animal Care and Use Committee.

### Immunohistochemistry

Brains and tumors from all experimental mice were dissected and either frozen or preserved in 10% formalin For Histology brain and tumors from BI6727-treated and vehicle-treated mice were harvested, rinsed in PBS, and fixed in 4% paraformaldehyde overnight at 4°C, and embedded in paraffin. Antigen retrieval was performed by application of citrate buffer pH 6.00 for 20 minutes. Slides were then incubated with anti INI1-BAF47, anti-Ki67 (Lab Vision; SP6 RM-9106-S) cleaved caspase 3 and H&E overnight at 4). and secondary antibody conjugated to horseradish peroxidase was applied and detected using the Dako Envision Kit for 3,3′-diaminobenzidine.

## SUPPLEMENTARY MATERIALS FIGURES


