# Supplementary material for: Targeting Polo-like kinase 1 in SMARCB1 deleted atypical teratoid rhabdoid tumor
Source: Oncotarget. 2017 Oct 19;8(57):97290–303. doi: 10.18632/oncotarget.21932 (PMC5722562; doi:10.18632/oncotarget.21932)
Supplement: Supplementary file 1 [file oncotarget-08-97290-s001.pdf]

Targeting Polo-like kinase 1 in SMARCB1 deleted atypical teratoid rhabdoid tumor

SUPPLEMENTARY MATERIALS

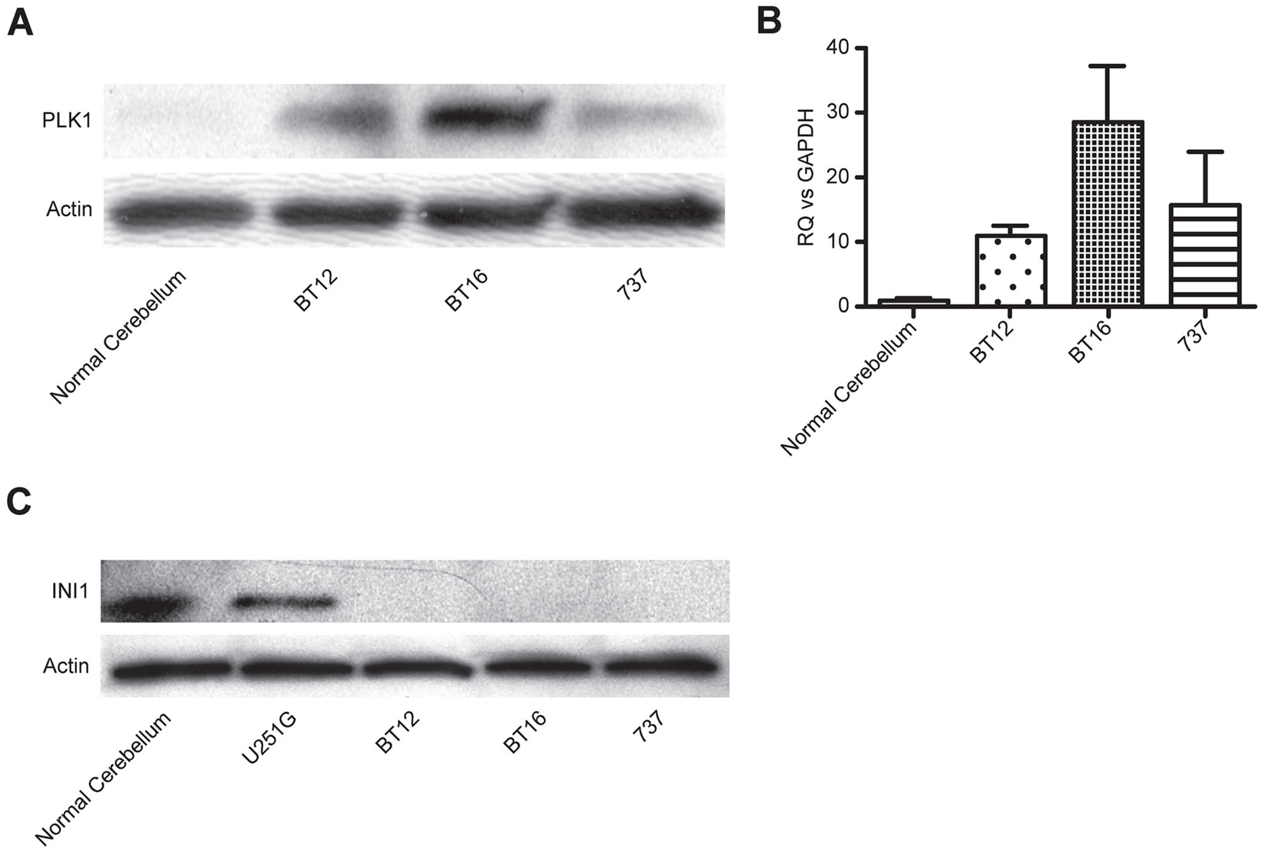

Supplementary Figure 1: Expression of PLK1 in ATRT cell lines compared to normal cerebellum: elevated levels of PLK1 in pediatrics INI1 deleted ATRT cell lines. (A) PLK1 protein expression by western blotting in the three ATRT cell lines compared to protein lysates from normal cerebellum. **(B)** PLK1 mRNA expression by qRT-PCR. Error bars represent standard error of the mean (SEM). **(C)** Expression of INI1 protein in normal brain, in GBM cell line U251G, and ATRT cell lines (BT12, BT16, and MAF-A737).

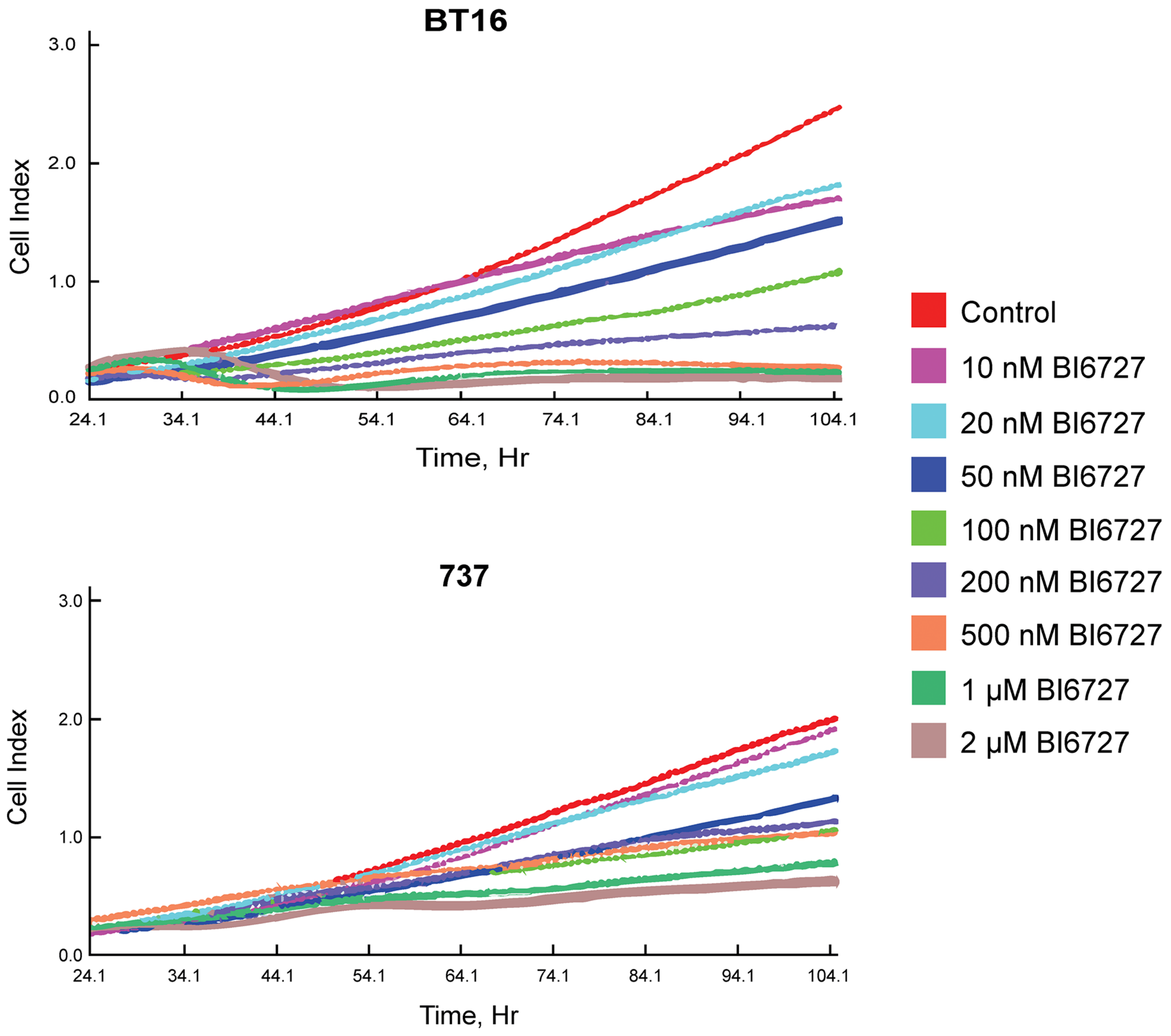

Supplementary Figure 2: Effect of BI6727 on ATRT cell proliferation by XCELLigence assay: BI6727 showed anti-proliferative effect from lower nanomolar concentrations. Both BT16 and MAF-A737 cells were treated with their corresponding IC_{50} of BI6727 or vehicle and real time cell proliferation was monitored over 100 hours after treatment. There is a marked decrease in cell proliferation even at lower nanomolar concentrations of the drug BI6727 compared to DMSO-treated cells equivalent to the highest concentration of the drug.

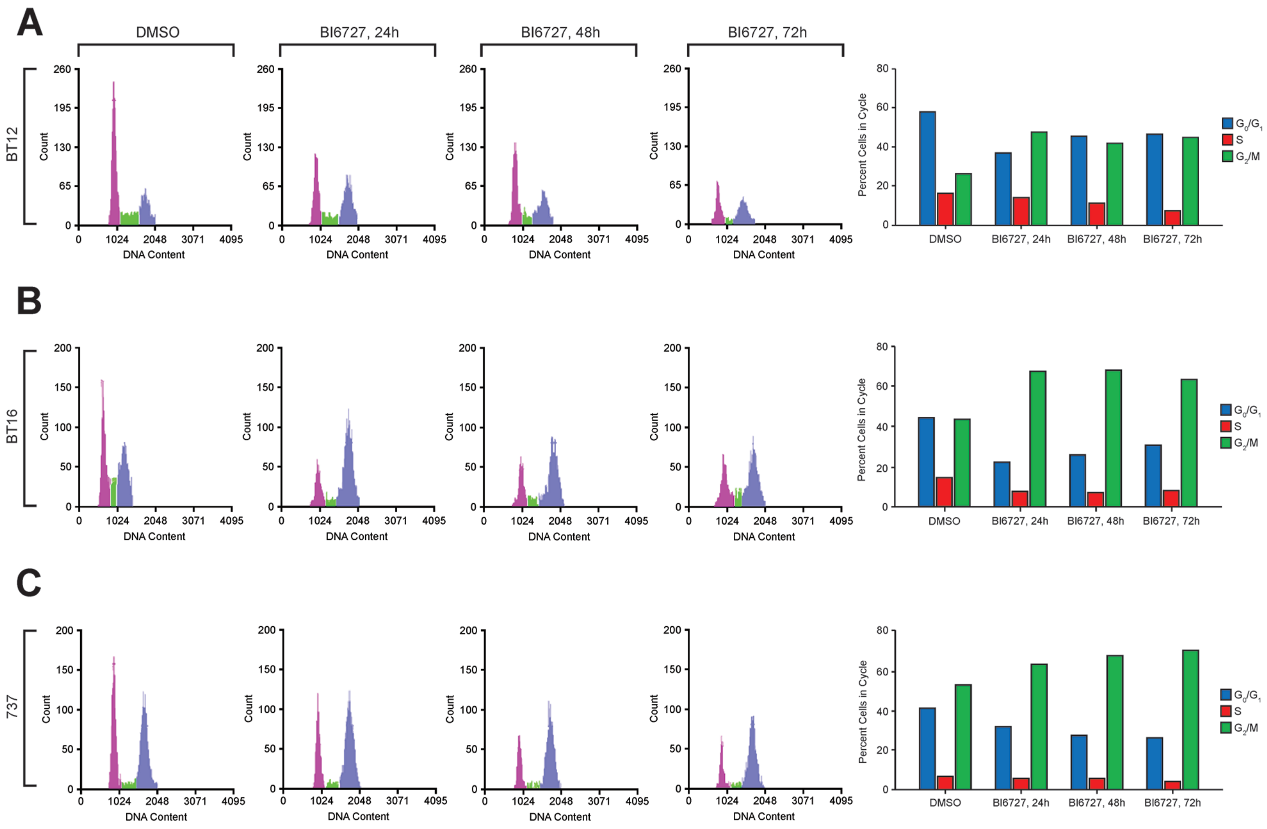

Supplementary Figure 3: Flow cytometric analysis of cell cycle distribution of BI6727 treated ATRT cell lines: consistent increase in G2/M phase of the cell cycle in all three ATRT cell lines. ATRT cells treated with IC_{50} of BI6727 for different time intervals. Representative bivariate DNA-counts cell cycle distribution of BT12 (**A**), BT16 (**B**) and MAF-A737 (**C**) were shown. The three discrete populations of cells representing Go/G1, S and G2/M areas were shown. The quantitation of cells in the three areas is given in the adjoining bar graphs.

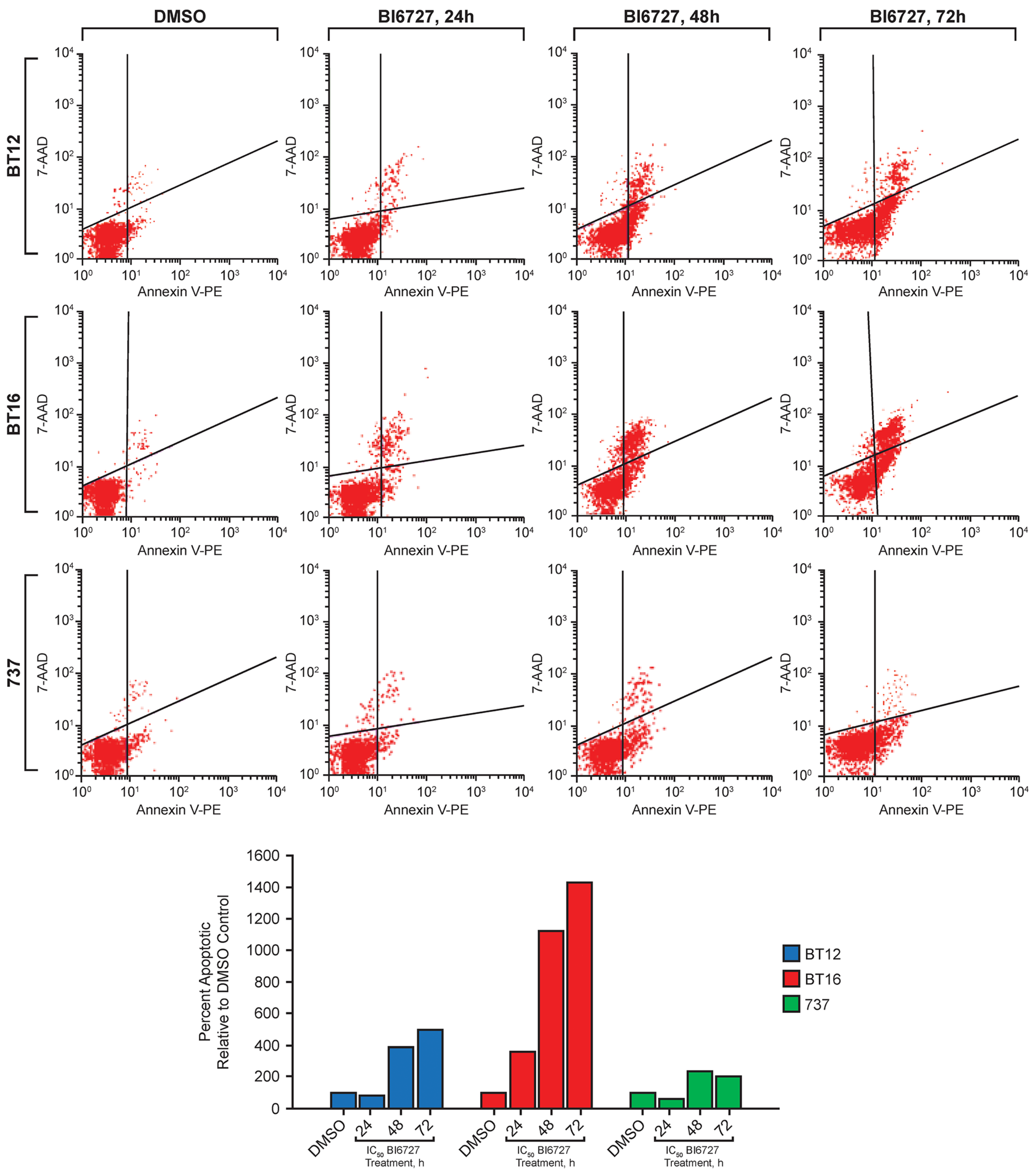

Supplementary Figure 4: Flow cytometric evaluation of apoptosis and ATRT cell viability with BI6727 treatment: a significant increase in cell apoptosis observed. (**Top**) Annexin V and 7-AAD staining of unfixed cells: Control, DMSO treated cells predominantly annexin V-negative and 7-AAD –negative staining (lower left quadrant). All BI6727-treated cells showed additional cell population with higher annexin staining denoting increase in cell death. (**Bottom**) Quantitation of percent apoptotic cells in BI6727 treated cells compared to DMSO control in the three ATRT cell lines shown.

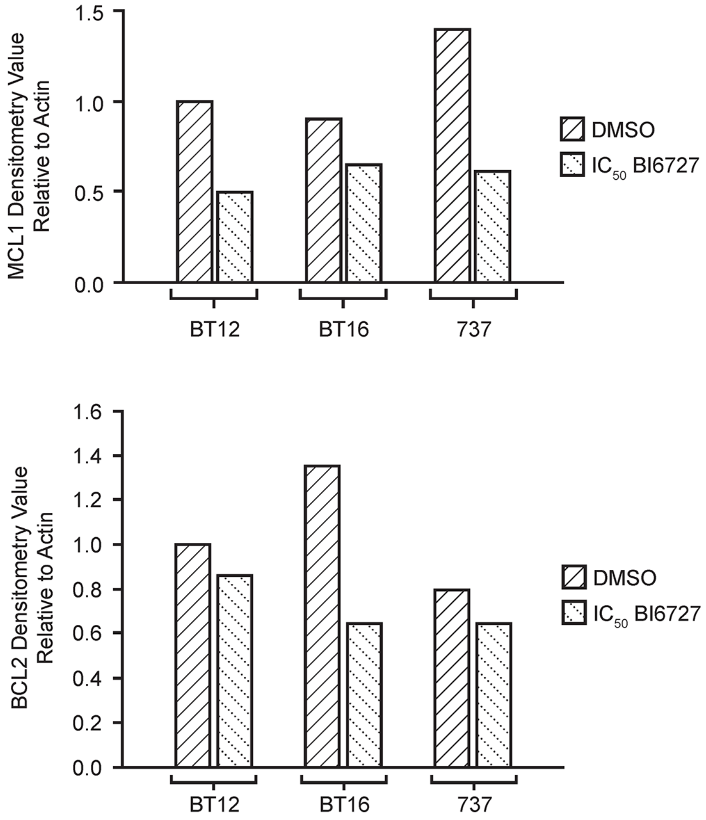

Supplementary Figure 5: Western blot quantitation for protein levels: decrease in both anti-apoptotic proteins MCL1 and BCL2. Using ImageJ, densitometric analysis was performed for MCL1 and BCL2 protein expression from western blots of cell lysates, normalized to that of actin and plotted. Bars with solid line: DMSO and dotted line: IC_{50} of BI6727 treatment.

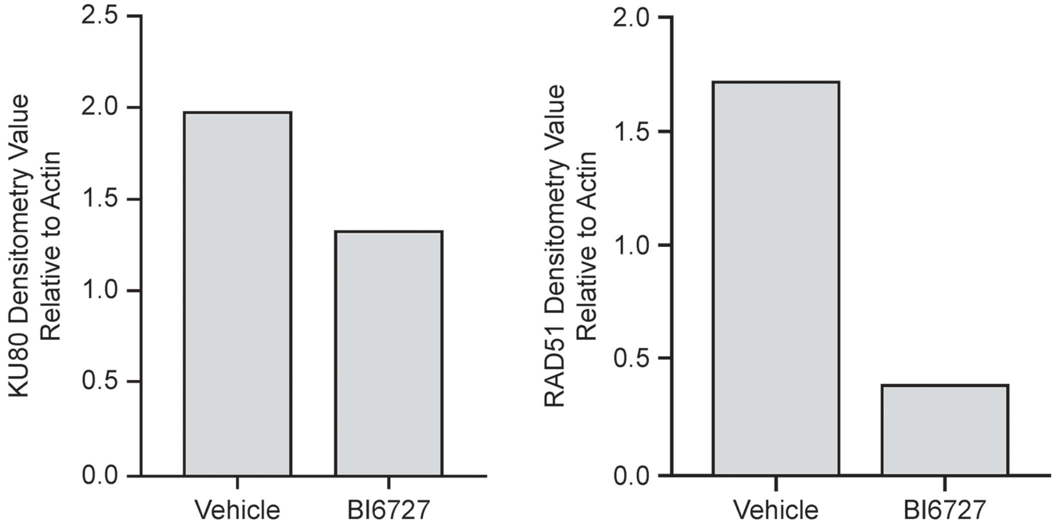

Supplementary Figure 6: Western blot quantitation for protein levels in BT16 xenograft tumors: decrease in both KU80 and RAD51. Using ImageJ, densitometric analysis was performed for KU80 and RAD51 protein expression from the lysates collected from mouse BT16 xenograft tissues, normalized to that of actin and plotted.

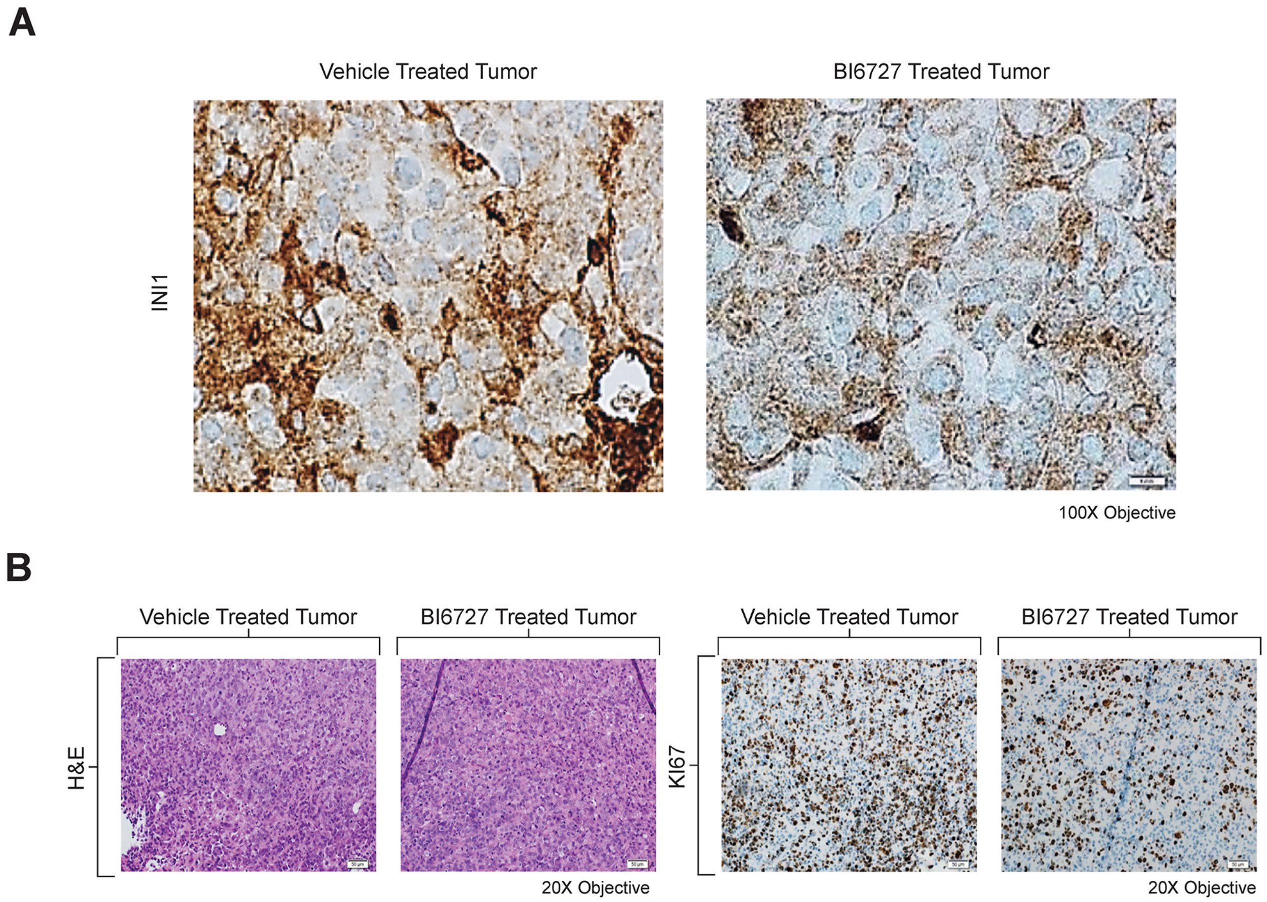

Supplementary Figure 7: Immunohistochemistry of intracranial tumors: validates INI1 deletion and a substantial decrease in tumorigenicity. (A) INI-1/SMARCB1 negative staining confirms SMARCB1-deletion in ATRT tumor. **(B)** Representative images of H&E staining confirm ATRT tumors. Accumulation of ki67, proliferation marker, decreased with BI6727 treatment *in vivo*.
